# Social visual preference mediates the effect of cortical thickness on symptom severity in children with autism spectrum disorder

**DOI:** 10.3389/fpsyt.2023.1132284

**Published:** 2023-06-16

**Authors:** Jierong Chen, Zhen Wei, Chuangyong Xu, Ziwen Peng, Junjie Yang, Guobin Wan, Bin Chen, Jianhua Gong, Keying Zhou

**Affiliations:** ^1^Department of Child Psychiatry and Rehabilitation, Affliated Shenzhen Maternity and Child Healthcare Hospital, Southern Medical University, Shenzhen, China; ^2^Key Laboratory of Brain, Cognition and Education Sciences, South China Normal University, Ministry of Education, Guangzhou, China; ^3^Center for Studies of Psychological Application, School of Psychology, South China Normal University, Guangzhou, China; ^4^Department of Child Health Care, Luohu District Maternal and Child Health Care Hospital, Shenzhen, China; ^5^Department of Pediatrics, Shenzhen People’s Hospital, The Second Clinical Medical College of Jinan University, First Affiliated Hospital of Southern University of Science and Technology, Shenzhen, Guangdong, China

**Keywords:** autism spectrum disorder, social visual preference, symptom severity, neuroanatomy, mediation

## Abstract

**Background:**

Evidence suggests that there is a robust relationship between altered neuroanatomy and autistic symptoms in individuals with autism spectrum disorder (ASD). Social visual preference, which is regulated by specific brain regions, is also related to symptom severity. However, there were a few studies explored the potential relationships among brain structure, symptom severity, and social visual preference.

**Methods:**

The current study investigated relationships among brain structure, social visual preference, and symptom severity in 43 children with ASD and 26 typically developing (TD) children (aged 2–6 years).

**Results:**

Significant differences were found in social visual preference and cortical morphometry between the two groups. Decreased percentage of fixation time in digital social images (%DSI) was negatively related to not only the thickness of the left fusiform gyrus (FG) and right insula, but also the Calibrated Severity Scores for the Autism Diagnostic Observation Schedule-Social Affect (ADOS-SA-CSS). Mediation analysis showed that %DSI partially mediated the relationship between neuroanatomical alterations (specifically, thickness of the left FG and right insula) and symptom severity.

**Conclusion:**

These findings offer initial evidence that atypical neuroanatomical alterations may not only result in direct effects on symptom severity but also lead to indirect effects on symptom severity through social visual preference. This finding enhances our understanding of the multiple neural mechanisms implicated in ASD.

## Introduction

Autism spectrum disorder (ASD) is a neurodevelopmental disorder characterized by persistent deficits in social interaction and the presence of restricted interest or stereotyped behaviors (1). The prevalence of ASD has increased sharply from 4/1,000 to 1/36 children in the past decade (2). The core symptoms of ASD typically manifest around the age of 2 years and are accompanied by developmental variations in brain structure, function, and connectivity that impact behavior throughout the lifespan (3). As a highly heritable disorder, the cause of ASD is complex and involved genetic and environmental factors, thereby presenting a significant challenge to understanding the pathology of this disorder (4). Notably, abnormalities in brain could represent a node resulting from these diverse contributing factors leading to the manifestation of ASD. Despite being recognized as a brain-based disorder and having undergone numerous neuroimaging studies, the underlying neural mechanisms of ASD remain incompletely understood.

Numerous neuroimaging studies have consistently identified a robust relationship between neuroanatomical alteration and autistic symptoms in individuals with ASD. For example, a study by Bedford et al. (5) reported greater cortical thickness in widespread brain regions in ASD aged 2–65 years, with this greater cortical thickness being positively correlated with calibrated severity scores (CSS) of the Autism Diagnostic Observation Schedule Version 2 (ADOS-2). Similarly, a large-scale analysis of structural MRI found that individuals with ASD aged 6–65 years exhibited significant positive correlations between ADOS scores and volumes of both gray and white matter, in addition to cortical surface area (6). Moreover, a comprehensive review of neuroanatomy in ASD suggested that there exists a robust association between the neuroanatomical foundations of ASD and the functional impairments that are typical of the clinical ASD phenotype (7). Recent study found morphological connectivity abnormalities in cortico-striatum-thalamic-cortical network can predict the severity of social communication deficits in young children with ASD aged 2–8 years (8). Functionally, brain regions that are related to symptom severity serve as neural network hubs and play crucial roles in social cognition and behavior (3). Although the link between brain structure and symptom severity in ASD has been widely established, the mechanisms underlying this relationship have yet to be fully elucidated.

Over the past decades, the characteristic visual preference in ASD has received increased attention from researchers. Most studies examining visual preference via eye-tracking have reported that individuals with ASD show reduced attention to social stimuli (e.g., human face and biological motion) and attention bias to non-social stimuli (e.g., geometric patterns and wheel) (9–11). This characteristic visual preference is defined as social visual engagement difficulties and is considered a potential early biomarker of ASD (12–15). This innate mechanism (i.e., social visual engagement) ensures that typically developing infants exhibit attention bias for social information (16), but it is pathognomonically impaired in children with ASD (17). Owing to dysfunction of this mechanism, individuals with ASD present a general lack of attention to the social environment, which may relate to the deficits of social-communicative functional circuits and altered brain structure (15, 18). For instance, a previous neuroimaging study found neural functional disconnection between the visual and attention networks and social brain networks in ASD subtypes with pronounced social visual engagement difficulties, while increased hypoconnectivity of the default mode network-occipito-temporal cotex was related to increased symptom severity (19). Consistent with this, structural neuroimaging studies in typically developing populations have demonstrated that neuroanatomical alterations in specific brain regions, such as the amygdala, fusiform gyrus (FG), and superior temporal gyrus, are correlated with social attention (20). Furthermore, the abnormal neuroanatomy and activation of these brain regions, which are key components of social functioning, have been suggested as being linked to symptoms of ASD (21, 22). Previous studies have also reported that toddlers with ASD who strongly preferred geometric images demonstrated decreased intellectual development levels, social skills, and more severe symptoms (23, 24). Taken together, these findings suggest the possibility of interactions among visual preference, brain structure, and clinical symptoms in ASD.

To our knowledge, prior research has not yet explored the relationship among these three factors. Nevertheless, based on a synthesis of previous research, we infer that there may be a mediating relationship among the three factors. Firstly, a well-established brain-symptom relationship exists where specific brain regions mediate core symptoms of ASD, especially social brain (25). Secondly, as previously noted, social visual engagement difficulties was mediated by specific brain regions, such as the amygdala (26), the temporal-parietal junction (27), the insula (28), and the FG (29). Thirdly, social attention deficits appear before core symptoms emerge during infancy. These early deficits may have a cascading effect on the development of social communication skills. For example, a longitudinal study for ASD children found the preference for biological motion at the age of 3 strongly predicted a reduction in severity scores on the ASD-G 1 year later (30). Similarly, the pattern of visual preference measured by the Geopref Test in individuals with ASD aged 1–3 years predicted ADOS total scores at school age (31). Jones and Klin (17) reported that infants who were later diagnosed with ASD exhibited typical eye gaze behavior (preferential attention to others’ eyes) at 2 months of age, but showed a significant decline from 2 to 24 months of age, in contrast to typically developing infants. The authors postulated that while reflexive social visual engagement is intact at birth for infants with ASD, the emergence of experience-dependent, cortically-mediated, spontaneous attention underlies the decreased social visual engagement observed in this population (17). Given the evidence of the independent impact of both brain development and symptom severity on visual attention in ASD, we propose social visual preference is a potential mediator of the brain–symptom relationship.

In the current study, we measured characteristic visual preference using the GeoPerf Test and social symptom severity, as assessed by the Autism Diagnostic Observation Schedule- Generic (ADOS-G) in children with ASD. Then, we used structural magnetic resonance imaging (sMRI) to determine structural differences between ASD and typical developing (TD) peers. Finally, we performed mediation analysis to explore whether social visual preference mediates the association between brain structure and symptom severity. Our hypotheses were as follows: (1) individuals with ASD would spend more fixation time on digital geometric images (DGI) than digital social images (DSI); (2) compared to TD, ASD participants would exhibit atypical cortical morphometry in specific regions, particularly social brain regions; (3) fixation percentage of DSI/DGI would be related to altered brain structure and symptom severity; and (4) social visual preference would mediate the relationship between brain structure and social symptom severity.

## Materials and methods

### Participants

Forty-six children with ASD and 28 TD children were recruited. All participants with ASD were selected according to the following criteria: (1) aged between 2 and 6 years; (2) diagnosed by experienced child psychiatrists, meeting Diagnostic and Statistical Manual of Mental Disorders (DSM-5) diagnostic criteria; and (3) having no history of seizures, neurological issues, head injury, or loss of consciousness. Inclusion criteria for TD participants included not having a history of developmental or neuropsychiatric disorder, either now or in the past, as well as having a gender and chronological age matching those of the ASD group. Individuals with a family history of any neuropsychiatric disorder, including ASD, learning disabilities, affective disorders, schizophrenia or epilepsy, were excluded from the study. Written informed consent was obtained from the parents of all children who participated in this study. Demographic features of the participants are provided in Table 1. There was no community involvement in the reported study.

**TABLE 1:** Demographic and clinical characteristics of ASD and TD participants.

	ASD	TD	*p*
Number	43	26	
Gender (male: female)	34:9	19:7	0.5676
Age	4.12 ± 1.28	4.23 ± 1.31	0.7327
ADOS-C	6.60 ± 1.54		
ADOS-SI	9.18 ± 1.87		
ADOS-SA	15.78 ± 2.95		
ADOS-CSS-SA	8.19 ± 1.48		
Fixation Time in DGI (ms)	1996.75 ± 1016.75	1459.63 ± 431.76	0.046
Fixation DGI (%)	40.76 ± 19.14	32.90 ± 11.44	<0.001
Fixation Time in DSI (ms)	1412.48 ± 912.70	2377.25 ± 849.53	<0.001
Fixation DSI (%)	28.68 ± 17.80	45.20 ± 15.77	<0.001
GDDS-Gross motor	61.51 ± 12.86		
GDDS-Fine motor	54.05 ± 16.23		
GDDS-Adaptive	49.91 ± 15.19		
GDDS-Language	39.51 ± 14.79		
GDDS-Social	46.15 ± 13.51		

Data are presented as mean ± SD. ADOS, autism diagnostic observation scale; ADOS-SI, social interaction score of autism diagnostic observation scale; ADOS-C, communication score of autism diagnostic observation scale; ADOS-SA, social affect score of autism diagnostic observation scale; ADOS-SA-CSS, Calibrated Severity Scores for the Autism Diagnostic Observation Schedule-Social Affect; DGI, dynamic geometric images; DSI, dynamic social images; GDDS, Gesell Developmental Diagnosis Schedules; % DGI, percentage of fixation time in digital geometric images; % DSI percentage of fixation time in digital social images.

### Clinical evaluation

Autism spectrum disorder participants received both Gesell Developmental Diagnosis Schedules (GDDS) and ADOS-G administered by a trained and experienced clinician.

The GDDS is used to assess the developmental level of children with ASD aged between 0 and 72 months; it is more applicable to toddlers and children with neurodevelopmental disorders than other scales (32). The results are expressed in terms of developmental quotients, with scores 86 or above being typical development, 75–85 indicating borderline development, 55–74 indicating mildly developmental delay, 40–54 indicating moderately developmental delay, 25–39 indicating severely developmental delay, ≤24 indicating extremely severely developmental delay.

The ADOS-G is a semi-structured, standardized assessment tool for individuals with suspected ASD across a wider developmental and age range (33). Based on the age and language of the participants, ADOS-G consists of four modules. In the present study, 33 ASD children received Module 1, 8 ASD children received Module 2, and 2 children received Module 3. This is a standardized instrument used to assess the communication and social interaction abilities of individuals with ASD. In the present study, we obtained scores for social interaction (ADOS-SI), communication (ADOS-C), and social affect (ADOS-SA). ADOS-SA is the sum of ADOS-C and ADOS-SI. To enable statistical pooling of ADOS scores across modules, raw ADOS scores for social affect (ADOS-SA) were converted into ASD severity scores calibrated to ADOS (ADOS-SA-CSS). These scores range from 1 to 10 and reflect the overall severity of ASD-related behavioral characteristics across the social interaction and communication domains (34).

### Eye-tracking paradigm

We produced an eye-tracking paradigm with reference to the Society GeoPref test (19, 35, 36). The paradigm consisted of DGI and DSI placed side-by-side in the scene changing simultaneously, without audio information. The video includes six individual scenes with a total of 60 s (each scenes displayed for 10 s) for the DGI and DSI (left/right) with random scene assignment across subject and diagnosis (Supplementary Figure 1). In this study, we employed the SMI RED250 portable eye-tracking system. The screen resolution was set to 1,024 × 768 pixels, with a spatial resolution of 0.03 degrees and a sampling frequency of 250 Hz. Children were seated in a dark, soundproof room with their parents, facing a 15-inch widescreen LCD monitor. The center of their gaze was aligned with the monitor’s center, and the distance between the eyes and the monitor was kept at 65 cm. Prior to the presentation of the short video stimulus, eye position correction was performed by instructing participants to focus on a dynamic pink rabbit. Eye-tracking data were collected while participants viewed these videos with two-eye tracking and five-point calibration at 250 Hz sampling rate. Gaze patterns were recorded using the BeGaze data analysis software system. GeoPref test includes three areas of interest (AOIs)—DGI, DSI, and background (Supplementary Figure 2). To determine the percentage of time spent on each AOI (i.e., “% DGI” and “% DSI”), the sum of fixation time for each AOI was divided by the total sum of fixation time for all three AOIs.

### Acquisition of MRI data

High-resolution anatomical images were obtained using a Siemens Prisma 3.0T (Siemens Medical Solutions, Erlangen, Germany) with a Siemens 12-channel receive-only head coil and a T1-weighted inversion recovery fast spoiled gradient-echo sequence. Foam padding was used to minimize head movement for all participants. Prior to the child entering the MRI scanner, a parent or legal guardian signed a consent form and remained present in an adjacent waiting room during the entirety of the scanning procedure. During scanning, the imaging data of TD children were collected during natural sleep at night, and Children with ASD were given sedation using 50 mg/kg of chloral hydrate (CH) in accordance with a strict clinical protocol established by the Radiology Sedation Committee of the hospital. It has been reported that mild to moderate doses of CH do not necessarily affect neural responses, and a maximum dosage of 75 mg/kg is set to minimize unwanted side effects while ensuring an appropriate level of sedation (37, 38). Therefore, CH may not disturb these findings in current study. High-resolution magnetic resonance (MR) images were acquired using a 3D T1 sequence with the following parameters: echo time of 3.30 ms, repetition time of 10 ms, flip angle of 15°, acquisition of 180 slices, and a voxel size of 1 mm × 1 mm in-plane. Two authors (JC and CX) manually inspected each raw MRI data for motion artifacts. After visual assessment of the MRI data and FreeSurfer output, three participants with ASD and two TD participants were excluded due to insufficient quality. Therefore, the final analysis included data from 43 participants with ASD and 26 participants with TD.

### Processing of MRI data

T1-weighted images were processed using FreeSurfer image analysis suite version 6.0.0.^1^ Eighty-three regions of interest, comprising forty-one areas in each hemisphere and an additional region in the brainstem, were defined using the Desikan–Killiany Atlas (39). The technical details of these procedures have been well documented in prior publications (40, 41). After the 3D surface was constructed, the cortical thickness was measured as the shortest distance from the white surface to the pial surface at each surface vertex. Cortical volume was measured by the volume of gray matter located between the white and pial surfaces. The surface area was measured by assigning an area to each vertex equal to the average of its surrounding triangles on the white surface. The cortical thickness, volume, and surface area were smoothed using a 10 mm full width at half maximum (FWHM) two-dimensional Gaussian kernel.

### Statistical analyses

Considering that numerous studies have provided evidence that neuroanatomical abnormalities in ASD are highly age- and gender-dependent, age and gender are suitable to be used as covariates in subsequent analyses (42, 43). Total cortical volume was also included as a covariate in subsequent analysis. A two-step general linear model (GLM) was employed to estimate differences in cortical thickness, volume, and surface area between individuals diagnosed with ASD and TD. To minimize false-positive results, a stringent criterion was applied, setting all analyses at *p* < 0.005 after correction for multiple comparisons using Monte Carlo simulations (44). The clusters that showed group differences in thickness, area, and volume after correction were selected as regions of interest (ROI). ROI values were then extracted for the follow-up analyses. A Fisher transformation was performed to enhance the normality of the correlation coefficient (45). Based on the extracted values of the ROI (i.e., mean, standard deviations, and numbers of participant), we used formulas provided by Lipsey and Wilson (46) to calculate the effect sizes and confidence intervals between the two groups (46).

Further analyses were performed using SPSS version 20 (IBM Corp., Armonk, NY, USA). Differences between % DSI and % DGI in the ASD group were assessed using a paired *t-test*. An independent *t-test* was performed to compare % DSI or % DGI between the two groups. Partial correlation analyses were conducted to explore the relationships among visual preference (% DSI or % DGI), brain structure, and symptom severity, while age and gender were used as covariates in the ASD group. A false discovery rate (FDR) correction for partial correlation was applied, and FDR-corrected *p-*values of < 0.05 were considered statistically significant. The % DSI related to the values of ROI and score of the ADOS-CSS-SA was then used to test the hypothesis that the relationship between altered brain structure and symptom severity is mediated by atypical visual preference. The present study used a script written by Hayes (47) to conduct mediation analysis using SPSS. The analysis was performed using the bootstrapping technique, which involves resampling to obtain confidence intervals for the mediator’s indirect effect. We obtained bias-corrected bootstrap 95% confidence intervals by generating 5,000 bootstrapped samples. Model four of PROCESS macro was selected. In the mediation model, % DSI was set as the mediator (M), ADOS-CSS-SA was set as the outcome (Y), the values of ROI were set as the predictor (X), and age and gender were set as covariates. All reported probabilities (*p-values*) were two-tailed, and values < 0.05 were considered statistically significant.

## Results

As detailed in Table 1, DGI attracted significantly more fixation time than DSI (*t* = 2.826, *p* = 0.006, Cohen’s *d* = 0.6095) in individuals with ASD. Compared to TD children, children with ASD spent more time fixating on DGI (*t* = 2.040, *p* = 0.046, Cohen’s *d* = 0.4713) and less time fixating on DSI (*t* = –3.695, *p* = 0.001, Cohen’s *d* = –0.9677). According to the result of GDDS, on average, children with ASD had mildly delayed development in gross motor skills, and moderately delayed development in language, adaptive, fine motor skills, adaptive functions, and social functions (Table 1).

**FIGURE 1:**
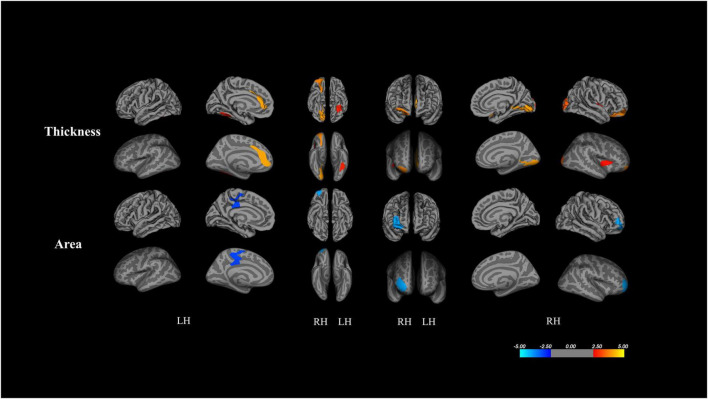
Results from whole-brain analysis of cortical metrics between ASD and TD peers, controlling for age and gender (corrected for multiple comparisons using Monte Carlo stimulations *p* < 0.005). Clusters surviving multiple comparison corrections are shown by red (ASD > TD) and blue (TD > ASD).

Figure 1 and Table 2 depict results from the whole-brain analyses comparing cortical thickness and surface area between the ASD and TD groups, after controlling for age and gender. There was no significant difference in volume between the two groups. Clusters surviving multiple comparison corrections are shown in red (ASD > TD) and blue (TD > ASD) in Figure 1. Children with ASD exhibited increased cortical thickness and smaller cortical area when compared to TD peers.

**TABLE 2:** Clusters of significant differences in cortical morphometry between TD and ASD.

Measurement		Region	Size (mm^2^)	x	y	z	CWP	NV	Cohen’s d	95% CI
Thickness	ASD > TD	Left Rostral Anterior Cingulate	1122.47	–7.3	37.5	13.5	0.0001	2270	1.0775	0.5523, 1.6026
		Left Fusiform	687.19	–35.5	–44.1	–21.1	0.0035	1181	1.3755	0.831, 1.92
		Right Lingual	1946.68	16.7	–65.3	–3.6	0.0001	2351	1.6948	1.1317, 2.2579
		Right Rostral Middle Frontal	1005.30	20.0	50.4	–13.4	0.0002	1625	0.9048	0.395, 1.416
		Right Lateral Occipital	909.12	27.3	–93.1	4.6	0.0006	1154	0.7224	0.2208, 1.224
		Right Insula	715.56	37.5	–2.7	1.6	0.0036	1892	1.3368	0.8012, 1.8724
Area	ASD < TD	left Paracentral	973.87	–17.1	–30.1	43.1	0.0015	2670	1.1769	0.6432, 1.7106
		Right Rostral Middle Frontal	1332.19	27.7	57.8	–9.5	0.0001	1849	0.9599	0.4388, 1.481

Results from whole-brain analysis of cortical metrics between ASD and TD peers, controlling for age, gender, and total cortical volume (corrected for multiple comparisons using Monte Carlo stimulations p < 0.005). CWP, Cluster-Wise P-value; NV, number of vertices; CI, confidence intervals.

**FIGURE 2:**
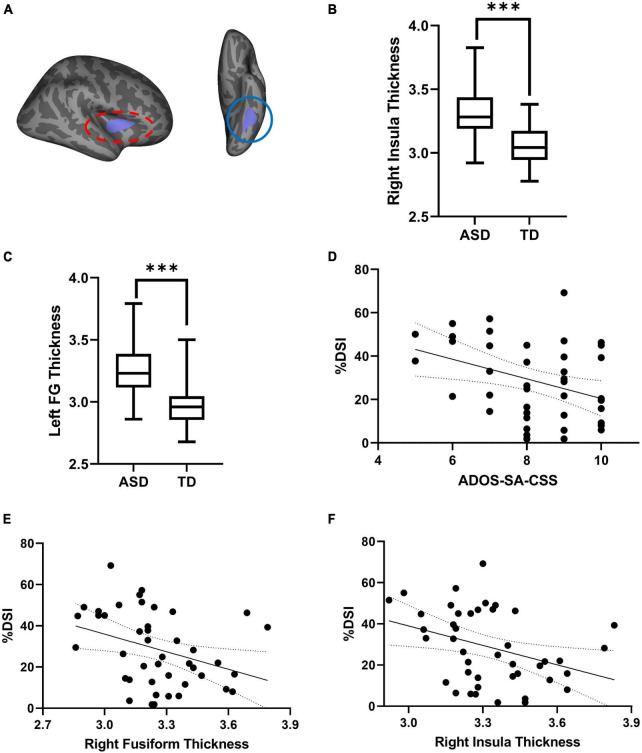
**(A)** Cortical thickness of left FG (red oval) and right insula (blue oval) exhibiting significant differences between TD and ASD. **(B,C)** Comparison of thickness in left FG and right insula between two groups. **(D)** Scatterplot of the correction between %DSI and ADOS-SA-CSS. **(E,F)** Scatterplot of the correction between thickness and %DSI. ADOS-SA-CSS, calibrated severity scores for the autism diagnostic observation schedule-social affect; %DSI, percentage of fixation time in digital social images. ^***^p < 0.005.

As shown in Figure 2 and Table 3, the partial correction analyses revealed that ADOS-SA-CSS correlated with % DSI (*r* = –0.512, *p* = 0.014), left FG thickness (*r* = –0.393, *p* = 0.046), right insula thickness (*r* = –0.505, *p* = 0.014), and right rostral middle frontal thickness (*r* = –0.417, *p* = 0.041). We also observed that % DSI was correlated with % DGI (*r* = –0.462, *p* = 0.024), left FG thickness (*r* = –0.496, *p* = 0.014), and right insula thickness (*r* = –0.442, *p* = 0.030). The *p-*values of the partial correction were corrected by FDR. Because % DSI was related to brain structure and symptom severity, we selected it as the mediating variable.

**TABLE 3:** Partial correlation among ADOS total score, fixation time, and cortical metrics in ASD.

	ADOS-SA-CSS	Fixation DSI (%)	Fixation DGI (%)
Fixation DSI (%)	–0.512*	–	–
Fixation DGI (%)	0.158	–0.462*	–
Left Fusiform Thickness	0.393*	–0.496*	0.130
Right Insula Thickness	0.505*	–0.442*	0.228
Left Rostral Anterior Cingulate	0.166	–0.361	0.76
Right Lateral Occipital Thickness	0.291	–0.224	0.369
Right Rostral Middle Frontal Thickness	0.417*	–0.358	0.256
Right Lingual Thickness	0.299	–0.257	0.006
Left Paracentral Area	–0.049	0.142	–0.143
Right Rostral Middle Frontal Area	0.030	–0.024	0.07

Presented as partial correlation coefficient, control for age and gender; *p < 0.05, corrected for multiple comparisons using FDR. ADOS-SA-CSS, Calibrated Severity Scores for the Autism Diagnostic Observation Schedule-Social Affect; DSI, dynamic social images; DGI, digital geometric images; % DGI, percentage of fixation time in digital geometric images; % DSI, percentage of fixation time in digital social images.

**FIGURE 3:**
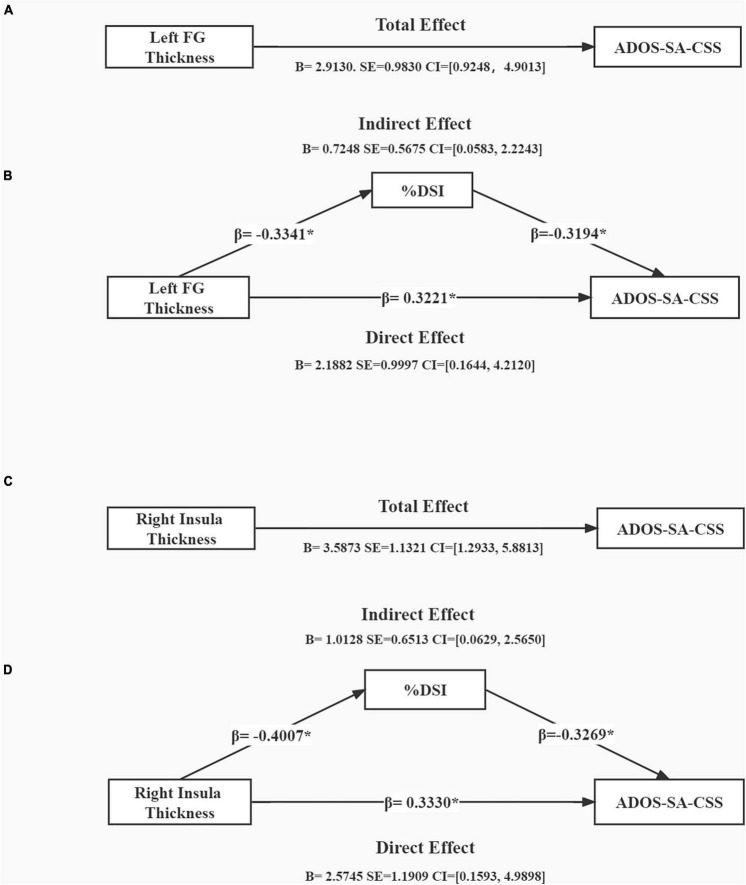
Mediation analysis showing total effect of cortical thickness on symptom severity **(A,C)** and indirect effect of %DSI **(B,D)**, control for age and gender. *p < 0.05. B, unstandardized regression coefficient; SE, standard error; CI, confidence interval; β, standardized regression coefficient; ADOS-SA-CSS, calibrated severity scores for the autism diagnostic observation schedule-social affect; %DSI, percentage of fixation time in digital social images.

We tested our mediation hypothesis using a mediation model, in which % DSI was postulated to mediate the relationship between brain structure (thickness of the left FG and right insula) and symptom severity (as measured by ADOS-SA-CSS) after controlling for gender and age (Figure 3). Figure 3A presents the mediation analysis showing the total effect of left FG thickness and ADOS-SA-CSS [*B* = 2.9130, SE = 0.9830, CI = (0.9248, 4.9013)]. Figure 3B shows that left FG thickness and ADOS-SA-CSS had a direct effect [*B* = 2.1882, SE = 0.9997, CI = (0.1644, 4.2120)]. The model showed a significant indirect effect of % DSI on left FG thickness and ADOS-SA-CSS [*B* = 0.7248, SE = 0.5675, CI = (0.0583, 2.2243)]. Figure 3C presents the mediation analysis showing the total effect of right insula thickness and ADOS-SA-CSS [*B* = 3.5873, SE = 1.1321, CI = (1.2933, 5.8813)]. Figure 3D shows that right insula thickness and ADOS-SA-CSS had a direct effect [*B* = 2.5745, SE = 1.1909, CI = (0.1593, 4.9898)]. The model showed a significant indirect effect of % DSI on right insula thickness and ADOS-SA-CSS [*B* = 1.0128, SE = 0.6513, CI = (0.0629, 2.5650)].

## Discussion

To our knowledge, this is the first study to explore a possible association between brain structures, visual preference, and clinical symptoms in children with ASD. First, we found that children with ASD presented more visual attention bias to non-social stimuli compared to TD children. Second, there were significant differences in cortical morphometry in children with ASD when compared to TD peers, including increased thickness and decreased area. Third, % DSI was negatively related to not only the thickness of the left FG and right insula, but also to ADOS-SA-CSS. Finally, a mediation analysis showed that % DSI partially mediated the relationship between neuroanatomical alternation (i.e., the thickness of the left FG and right insula) and symptom severity. These findings provide initial evidence that this brain-symptoms association occurs through mechanisms partially shared with social visual preference.

In the current study, children with ASD spent significantly more time viewing DGI than DSI. This is in congruence with a number of previous studies, in which individuals with ASD tended to show reduced attentional preference for social stimuli combined with a strong preference for and attention toward non-social stimuli (35, 48). The effect sizes were medium for visual preference between two groups. Similar to a spectrum, the pattern of visual preference in ASD would likely range from extreme preference for non-social stimuli to extreme preference for social stimuli (35). Based on this feature, recent studies have classified ASD participants into different subtypes according to the degree of visual preference. Specifically, these studies have found that preference for non-social subtypes predicts worse prognosis (19, 24). In addition, a significant negative association between ADOS-SA-CSS scores and % DSI was observed in children with ASD. Our results consistent with previous studies showing that visual preference in early childhood as a potential biomarker may predict symptom severity of ASD (23, 24). The findings from present study offer additional evidence for the distinctive visual preference observed in children with ASD that is linked to symptoms severity.

In the current study, we found differences in cortical morphometry between the ASD and TD groups. In line with prior research, we revealed that children with ASD exhibited a significantly thicker cortex in the frontal and temporal regions when compared to TD children (5, 49). Notably, these significantly different brain regions play an important role in cognitive and emotional processes (18, 50). In addition, Our findings partially align with previous research that reported decreased surface area in the frontal regions in individuals with ASD when compared with TD (51, 52). The current findings regarding brain volume corroborated earlier studies by showing no group differences in volume between ASD and TD at preschool age (49). Atypical thickness and surface area contribute to complex brain development trajectories of ASD in early life, which may be potential biomarkers and related to clinical symptoms (53, 54). Although the cause for cortical abnormalities in ASD is currently unknown, recent research suggests a strong correlation between transcriptionally downregulated genes associated with these abnormalities (55).

Increased cortical thickness in specific regions showed correlation with visual preference and symptom severity. Here, we found the right rostral middle frontal gyrus (rRMFG) related to ADOS-SA-CSS. The RMFG, a region implicated in phonology and semantic processing, contributes to the impairment of social communication (56). More importantly, the thickness of the left FG and right insula was related to symptom severity and % DSI, respectively. The FG is believed to be responsible for the ability to process facial features, making it a critical component for appropriate social interaction (57). However, in individuals with ASD, previous studies have shown abnormalities in FG structure and activation during face processing (58, 59). Previous studies have also demonstrated atypical activation of the insula in ASD during socioemotional processing tasks (60). Furthermore, dysfunction of the insula may be related to dysfunction of the broader salient network in individuals with ASD who do not find social stimuli salient and meaningful (61, 62). In line with previous study, Doyle-Thomas et al. (63) demonstrated that atypical morphometry in the FG and insula may be related to poorer social ability scores and greater social impairment. Although there was no significant correlation observed in the current study between visual preference and symptoms severity in other ROIs, we speculated other brain regions are involved in other dysfunctions and/or characteristics of ASD. Therefore, our findings further suggest that atypical morphometry in the FG and insula, which are the crucial neurological foundations of social information processing, appeared to be related to more severe symptoms and less social attention in children with ASD.

In addition, it is important to note that the FG and insula are especially critical for visual preference and symptom severity, and not the homologs or bilateral differences. One potential explanation is the altered structural brain asymmetry and lateralization observed in ASD. Dougherty et al. (59) showed atypical leftward asymmetry in FG structure, which is related to symptom severity in ASD. Functionally, the left and right FG are considered to perform distinct functions. The right FG is believed to be involved in conscious processing of faces, while the left FG engages more broadly in visual perception and object recognition (64, 65). Previous studies have shown that altered anterior insular asymmetry of ASD related to the scores of ADOS [(66, 67)]. Atypical insular asymmetry in ASD may contribute to the development of networks with a diminished salience signal to human faces and voices, and may lead to more learned passive avoidant responses to such stimuli (66). Our study adds evidence to support that atypical lateralization of special regions is related to symptom severity in ASD. As we know, there is little research to explore the relationship between visual preference and lateralization. Our findings at least lend support to the idea that structural asymmetries in the FG and insula are related to social visual preference and symptom severity in ASD.

Importantly, we have revealed social visual preference partially mediates the relationship between altered brain structure (the left FG and right insula) and symptom severity. Consistent with previous findings, abnormal thickening of gray matter in core brain regions can reduce visual preference for social stimuli (19, 27) and increase the severity of symptoms (68). Meanwhile, reduced visual preference for social stimuli can further exacerbate symptoms (69). These findings suggest the existence of a brain-trait (social attention)–symptoms pathway, which contributes to our understanding of one of the multiple neural mechanisms involved in ASD. Notably, our results also provide some clues for interventions. Our research findings support that social attention is influenced by core brain regions and also affects the manifestation of symptoms. We infer that social attention may be a promising intervention target for improving symptom severity by mitigating not only its direct effect but also the indirect impact of altered brain structure. To date, there have been no specific interventions for social visual preference. However, some special interventions have been developed based on the characteristic visual preferences of ASD, such as Lego^®^ Therapy (70) and The Transporter (71). These interventions created autism-friendly contexts mixed with social elements, which can not only attract the interest of children with ASD but also promote their spontaneous cognitive processing and learning. Moreover, Jones et al. (72) found early parent-mediated intervention has the potential to increase attention to social stimuli in infants at familial risk for ASD (72). Future research could develop interventions based on the characteristic visual preferences of ASD to increase interest and motivation for a better intervention effect.

Some limitations should be considered when interpreting our results. First, our study did not match the sample sizes of the ASD (*n* = 45) and TD groups (*n* = 26). Our future studies will recruit more TD children who meet these criteria. Moreover, there are close and interdependent relationship between brain structure and function. Our study revealed structural abnormalities in specific brain regions that appeared to be linked to functional abnormalities. However, sMRI does not adequately capture the temporal dynamics of brain function. Future studies could employ functional neuroimaging techniques, such as fMRI or functional near-infrared imaging technology, to explore related functional abnormalities. Furthermore, based on the findings of our present study, IFG and insula may serve as hubs for further functional analysis, which could help elucidate mediating mechanisms more enrichment and depth.

## Conclusion

In conclusion, this study demonstrated that social visual preference partially mediates the relationship between altered brain structure (in the left FG and right insula, specifically) and symptom severity. These findings offer initial evidence that atypical neuroanatomical alterations may not only result in direct effects on symptom severity but also lead to indirect effects on symptom severity through social visual preference. This finding enhances our understanding of the multiple neural mechanisms implicated in ASD.

## Data availability statement

The raw data supporting the conclusions of this article will be made available by the authors, without undue reservation.

## Ethics statement

The studies involving human participants were reviewed and approved by The Research Ethics Board of the Luohu District Maternal and Child Health Care Hospital. Written informed consent to participate in this study was provided by the participants’ legal guardian/next of kin.

## Author contributions

JC, ZW, JG, and KZ conceived the study, prepared the data, analyzed the data, and drafted and revised the manuscript. CX, BC, and JY helped to analyze the data and helped to draft the manuscript. GW and ZP participated in the study design and helped to draft and revise the manuscript. All authors approved the final version of the manuscript.

## Footnotes

1
http://surfer.nmr.mgh.harvard.edu/
