# Supplementary material for: Social visual preference mediates the effect of cortical thickness on symptom severity in children with autism spectrum disorder
Source: Front Psychiatry. 2023 Jun 16;14:1132284. doi: 10.3389/fpsyt.2023.1132284 (PMC10311909; doi:10.3389/fpsyt.2023.1132284)
Supplement: Supplementary file 1 [file Data_Sheet_1.docx]

**Supplementary Materials**


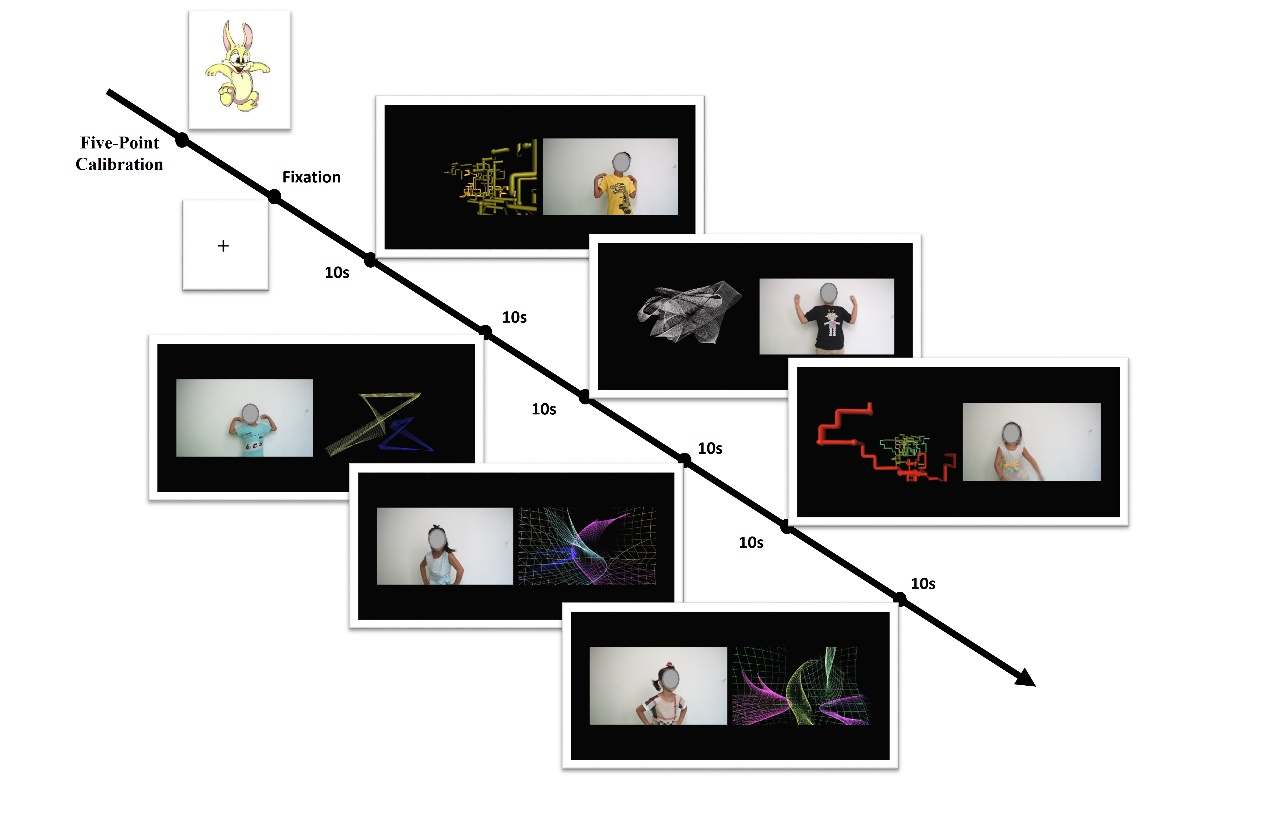


Figure 1 Eye tracking Paradigm of Society Geopref Test


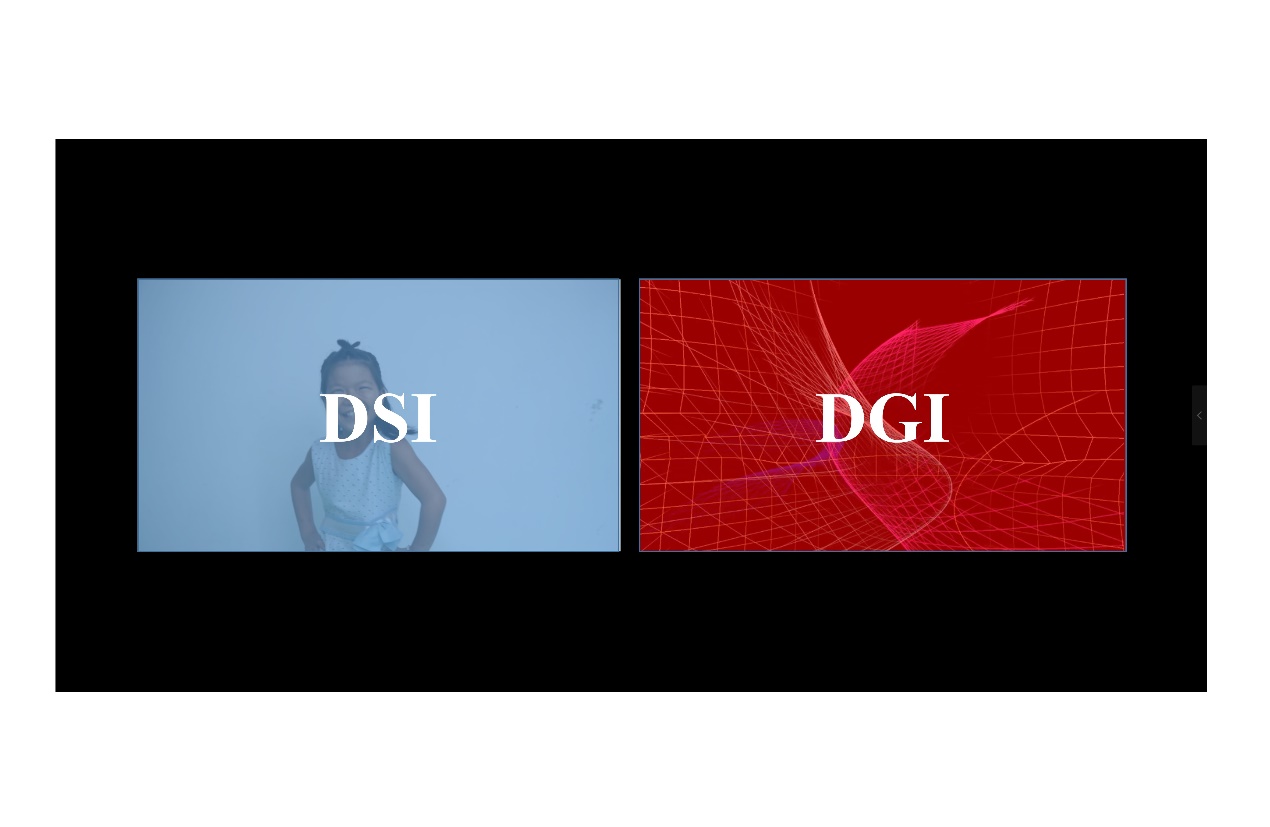


Figure 2 AOIs of DGI and DSI were identical in size (13.75 cm horizontal x 9.5 cm vertical; visual angles 12.91° horizontal and 9.05° vertical). The blue square region is AOI of DSI, the red region is AOI of DGI, and the remaining black area is the background. Abbreviation: DSI, digital social images; DGI, digital geometric images.
